# WHY IS THE CENTER OF EVIDENCE-BASED DERMATOLOGY RELEVANT TO INDIAN DERMATOLOGY?

**DOI:** 10.4103/0019-5154.53184

**Published:** 2009

**Authors:** Hywel Williams

**Affiliations:** *From the Centre of Evidence-Based Dermatology, King's Meadow Campus, University of Nottingham, Lenton Lane, Nottingham NG7 2NR, UK*

**Keywords:** *Evidence-based dermatology*, *India*, *knowledge*, *systematic reviews*, *trials*

## Abstract

Evidence-based dermatology is the application of high-quality evidence to the care of individual patients with skin diseases. The Centre of Evidence-Based Dermatology in the UK promotes activities in this field through its three interlinking cogs, composed of the international Cochrane Skin Group, the UK Dermatology Clinical Trials Network (UKDCTN), and the UK national electronic library for skin disorders. The Cochrane Skin Group summarises what is already known about health care interventions by supporting systematic reviews of relevant randomized controlled trials (RCTs). The UKDCTN then addresses the key research gaps identified in systematic reviews by coordinating and carrying out well-designed RCTs. The Skin Disorders specialist library then plays a key role in disseminating new knowledge from systematic reviews and RCTs to a community of clinical users. The electronic resources at the Centre of Evidence-Based Dermatology are all freely available to Indian Dermatologists who can use the resources in a way that could benefit their patients. Such new knoweldge only has value if it is shared and used.

## Rationale for this Article

**Figure 1:**
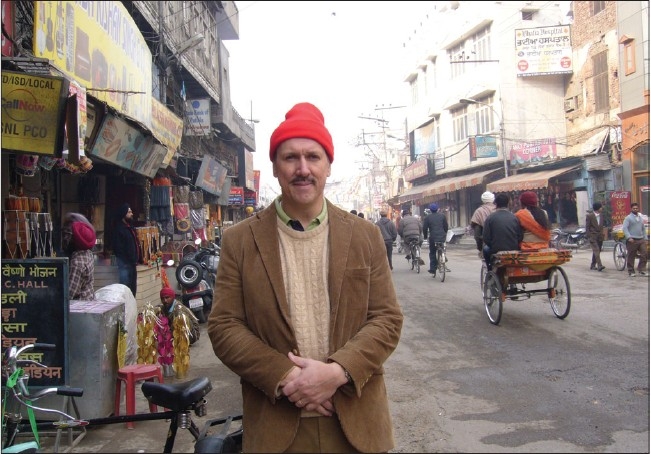
The author wearing a silly red hat in Amritsar

In 2008, it was a great honor for me to be invited by Dr. A.J. Kanwar and colleagues to visit the 36^th^ National Conference of the Indian Association of Dermatologists, Venereologists, and Leprologists in Chandigarh and to deliver some key lectures on the topic close to my heart – evidence-based dermatology. It was my first visit to India, and I found it a moving, humbling and enjoyable experience, and I am sure it will not be my last [Figure 1]. A number of colleagues came up to me after my talks to explore how the material was relevant to India. One of those was one of the editors of this journal, Dr. Saumya Panda. It was clear from talking to Dr. Panda that he immediately saw the need and potential benefits of promoting the principles of evidence-based dermatology in India, and it is through his kind invitation that I am sharing some preliminary thoughts with you.

This article is very much a personal reflection on how the work we do at the Centre of Evidence-Based Dermatology in the UK might be relevant and useful to colleagues in India. It is not a systematic review or an in depth critique of a body of literature, but simply a description of our work and how it might be applicable to dermatology, venereology, and leprology in India. I hope that the article does not sound patronising. There is one thing I have learnt from visiting other countries and that is that we share very similar problems – by that I mean our collective ignorance of how best to prevent and treat skin diseases. We all share the same challenge of translating new knowledge about skin disease treatment that is based on groups of patients participating in clinical studies to the particular circumstances of an individual with a skin problem who has come to you for help at that particular time. So rather than talk about countries and their differences, I am going to focus on what pulls us all together – our concern for improving the welfare of people with skin diseases.

## What is Evidence-based Dermatology?

### One world, not two

**Figure 2:**
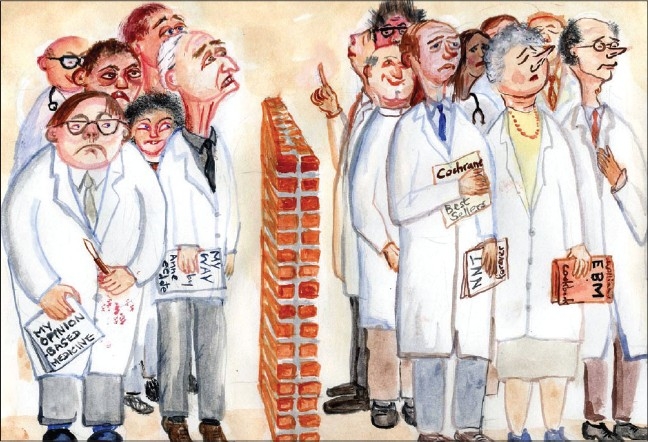
It is not as if there are two worlds in dermatology - one practicing evidence-based dermatology and other not, each separated by a brick wall. We are all practicing evidence-based dermatology to some extent, sometimes without knowing it (Picture drawn by the author)

Put simply, evidence-based dermatology is the application of external high-quality evidence to the care of individual patients.[1] It is a mistaken notion that the world of dermatology can be split into those who practice evidence-based dermatology and those who do not [Figure 2]. We are all practicing evidence-based dermatology to some extent since every doctor who cares for his or her patients will want to do the best for them by using treatments that work well and are safe. But like any other framework for understanding, the skills of formulating answerable questions based on clinical encounters, searching efficiently for the relevant evidence, critically appraising that evidence, and then interpreting and applying that evidence to a patient have to be learnt and practiced.

### The evidence hierarchy

**Figure 3:**
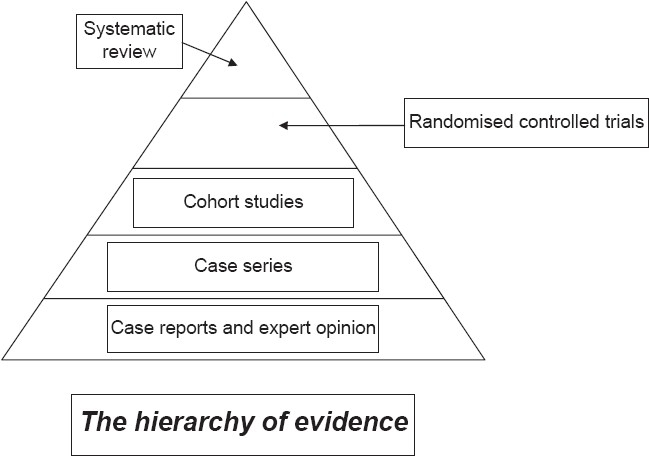
The evidence-based medicine hierarchy places systematic reviews of well conducted randomised controlled clinical trials at the top

Evidence-based dermatology implies a hierarchy of evidence that starts with systematic reviews at the top with anecdotes and case reports at the bottom [Figure 3]. It places considerable emphasis on the randomised controlled clinical trial (RCT) as the building block for informing clinical practice.[2] Such reliance on clinical trials has arisen because a well-conducted clinical trial offers the most reliable way of minimising bias when trying to answer a question on disease efficacy. Bias may be defined as a systematic error that results in the incorrect interpretation of a study result. Sometimes this is deliberate (for example, using a comparator drug at an inappropriate dose or frequency to make the new drug look better) and sometimes it is difficult to avoid (e.g., it is impossible to fully blind an RCT of oral retinoids when compared against placebo). RCTs are not the answer to everything and other study designs such as qualitative research surveys are needed to answer why some people with acne seek treatment and others do not. Surveys are needed to estimate the prevalence of filariasis, case control studies are needed to determine risk factors for recurrent cellulitis, and cohort studies are needed to determine the natural history of vitiligo in children.

### Minimising harm

Evidence-based dermatology is not a restriction on clinical freedom if one defines clinical freedom as the ability to do the best for one's patients as opposed to doing what you have been doing for the last 30 years simply because it has always been done that way. History is replete with examples of well-meaning doctors causing harm to their patients because of their failure to conduct RCTs and to then summarise those RCTs within systematic reviews. The use of oral corticosteroids for acute brain injury is a good example. The use of oral corticosteroids to reduce edema in acute brain injury was a standard practice until a very large trial showed that it was killing more people than it helped.[3] Dermatology is not immune from harming patients through misguided beliefs. Thus, mutilating operations such as 10 cm excision margins for primary cutaneous melanoma and radical vulvectomy for lichen sclerosus are now things of the past, yet they were practiced for decades by our predecessors on the basis of anecdote and case reports and a failure to question the rationale for the treatments we use.[4] The same applies to high-dose oral corticosteroids for treating pemphigoid or the use of thalidomide for toxic epidermal necrolysis, both of which have been shown to cause more harm than benefit when compared with potent topical corticosteroids or good supportive care respectively.[5,6] So evidence-based dermatology is every clinical dermatologist's business and not something that can be left to a few academics who may end up asking irrelevant study questions or promulgating lots of data that is clinically meaningless. We must all get involved in making sense of how to best treat patients and we should not be afraid of challenging current practices and changing our practice in light of new knowledge, regardless of how experienced we become. Our work at the Centre of Evidence-Based Dermatology can be considered as three consecutive processes: systematic reviews to summarise what is known, clinical trials to address knowledge gaps, and finally knowledge management and dissemination.

## Step 1: Summarising What is Known Through Systematic Reviews

### One RCT is never enough

Even though randomised controlled trials offer the best study design to minimise the risk of bias when evaluating the efficacy of a new treatment, one RCT is rarely enough for three reasons. The first is that a positive result could have simply arisen through chance. Given that most trial reports claim that the result is statistically significant if the *P*-value reaches the “magic” *P* = 0.05 level, it follows that around one in twenty trial reports reaching that level of evidence will falsely claim a real difference when none really exist. Therefore, with around over 17,000 trials relevant to skin already published, there will be a lot of studies out there that claim an effect when the results are really due to the play of chance. That is why the Food and Drug Administration (FDA) requires two identical trials to be conducted for licensing purposes for a new drug in order to reduce the chances of a single false positive result. The tendency to selectively publish positive studies (publication bias) means that the results of single trials, even when large and published in prestigious journals, are frequently contradicted when the totality of subsequent evidence is considered.[7] Conversely, there is a danger that many potentially effective therapies have been discarded in dermatology simply because the studies have been far too small and the authors and readers have mixed up inconclusiveness as being the same as not effective.[8] Systematic reviews that use the technique of meta-analysis to add together several similar but smaller inconclusive trials can sometimes produce an overall clear answer. The second reason why one RCT is not enough is because it is sometimes difficult to generalise from the people included in just one study. For example, a clinical trial of calcipotriol in a group of older men with plaque psoriasis living in Wales may contain a study population that is too different to inform treatment choices facing young women presenting with guttate psoriasis in Kolkata. A more generalisable answer about an intervention may be achieved by considering several studies that have been assembled together that include different populations, different ages, cultural beliefs, and co-morbidities. The final reason of why one RCT is never enough is that despite efforts to minimise bias, RCTs can be reported in misleading ways that can be influenced by hidden motives such as making profit for a company or for promoting individuals. Systematic reviews mitigate against such framing bias by drawing attention to possible conflicts of interest and by pointing out what was not said in the trial report as well as what was highlighted. A major part of systematic reviews is to draw attention to the quality of reporting of trials, and whether the outcomes were analysed in the way they were meant to be analysed. It is only by putting all relevant information together that we are best able to make sense of a particular intervention. Even if the conclusion of such a systematic review is simply to state that no reliable evidence has been produced to date to guide practice and that the best way forward is to start conducting well designed trials, it is a start in the right direction.

Traditional expert reviews might sound appealing, but they frequently fail to mention important studies and they may be prone to selective citation to support the expert's prejudice simply because they have not been done in a systematic and explicit way.[9] A systematic review on the other hand, follows an explicit process of formulating a precise question, searching exhaustively for all relevant information, quality appraising that information, sometimes combining that information in a meta-analysis if appropriate, and then interpreting and disseminating that information.

### The Cochrane Collaboration

**Figure 4:**
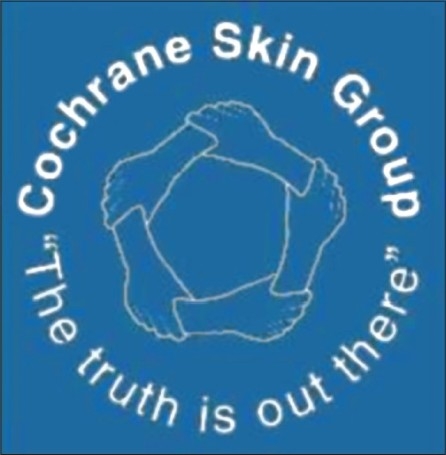
The Cochrane Skin Group logo shows five hands representing the five continents of the world joined together in the pursuit of the truth about skin disease treatments

It was the failure of the medical profession to systematically collate evidence from high quality randomised controlled trials that prompted the late Professor Archie Cochrane to challenge doctors to do something about it in the 1970s. The International Cochrane Collaboration was formed 25 years later in response to this challenge. The Cochrane Collaboration is a non-profit organisation of individuals that now contains over 50 review groups covering all aspects of human medicine. The Cochrane Skin Group [Figure 4] is one such group that was formed in 1997 and is supported by an editorial base at our Centre of Evidence-Based Dermatology in Nottingham. The editorial base comprises a review group coordinator, an editorial assistant, a trials search co-ordinator, and a co-ordinating editor (the author). Our job is to prioritise and support high-quality systematic reviews of interventions to prevent or treat skin diseases using the methods developed by the Cochrane Collaboration. So far, the Skin Group has published 39 full reviews and a similar number of published protocols that will eventually become full reviews. Some reviews relevant to dermatology are produced by other groups such as the Wounds, Pregnancy and Childbirth or Infectious Diseases Groups so that there are now over 100 reviews published on the *Cochrane Library* relevant to dermatology. Some have criticised Cochrane reviews for always concluding that there is insufficient evidence to recommend anything. Whilst this might be true for a lot of skin conditions where the evidence has been characterising by lots of small, poorly reported studies, a recent review found that Cochrane dermatology reviews fond that clear clinical recommendations were made in around 40% of cases.[10] Cochrane Skin Group reviews are of high relevance to people around the world and not just those who work in the UK. Not only do they deal with common conditions such as acne, psoriasis, and eczema, which affect all of the world's populations, but they also cover diseases that occur in developing countries such as cutaneous leishmaniasis, leprosy, and tropical ulcers. Venereal diseases are dealt with by the relatively newly formed Cochrane Sexually Transmitted Diseases group. Unlike other publications that quickly become out of date, Cochrane reviews are updated periodically as new evidence becomes available. Anybody can get involved in preparing and maintaining reviews but the work is hard and a degree of training and experience is needed before somebody leads a new review team. At the very least, anyone can read Cochrane reviews and use the evidence to inform daily practice. Cochrane reviews are published every quarter in an online product called the *Cochrane Library*, which also contains the largest database of clinical trials in the world and other useful methodological resources. And here is the most fascinating thing about the whole endeavour: During one of my talks at the 36^th^ National Conference of the Indian Association of Dermatologists, I asked members of the audience about the Cochrane Library. Several had heard of it but none claimed to have access to it. Yet 10 minutes before that lecture, I tried accessing the Cochrane Library from a terminal at the meeting by typing in www.thecochranelibrary.org and found full access to the Cochrane Library that was completely free. So there is no excuse for not at least looking at Cochrane reviews because the Indian Government has made it possible to gain full and free access from any computer with an internet connection in India.

## Step 2: Addressing Uncertainties Through Randomised Controlled Trials

### Insufficient evidence

Sometimes, Cochrane systematic reviews come up with clear clinical messages on what should or should not be used in clinical practice (e.g., minocycline has never been shown to be more effective than other tetracyclines for acne).[11] But more often than not, the lack of quantity and quality of evidence found is insufficient to make clear recommendations. Such a finding is quite helpful for the clinician, in that it at least reassures us that we are not missing some new important finding about treatments that we should be using in practice. It is also helpful to produce reviews that find insufficient evidence as it helps to draw a line in the sand that indicates a desperate need for a decent quality randomized controlled trial to address that area. The review can also suggest some priority to the questions that can be addressed by subsequent trials. Such is our philosophy at the Centre of Evidence-Based Dermatology – use systematic review to inform the need for clinical trials, which are then run by the UK Dermatology Clinical Trials Network after gaining suitable funding in open national competition from State funding bodies such as the National Institute for Health Research Health Technology Assessment Programme.

### Purpose of the UK dermatology clinical trials network

The UK Dermatology Clinical Trials Network (UK DCTN) was formed in February 2002 with the aim of conducting high quality, multi-center clinical trials that answer questions of importance to clinicians and patients. It involves a collaborative network of dermatologists, dermatology nurses, health services researchers, and patients throughout the UK and Southern Ireland. The Network is completely independent of the pharmaceutical industry. This should not be taken as meaning that the Network is against industry studies, but simply that dermatologists in the UK wanted to study questions that industry would never fund. These include trials of orphan skin diseases like pyoderma gangrenosum or studies of non-pharmacological interventions such as water softeners for childhood eczema or studies of low cost generic treatments like tetracyclines versus oral prednisolone for bullous pemphigoid – all funded studies that are currently being run through the UKDCTN. The UKDCTN is a registered charity that is run by a network manager, senior trials manager, and an administrative assistant. The organization is very democratic and accepts suggestions for new trials from all of its members. Those are then prioritized by a panel and developed into pilot studies and full funding applications. The UKDCTN took some time to get off the ground because it invested time in getting its structures and processes right, and now it is running five national studies [Table 1]. The UKDCTN is highly interdisciplinary and it works closely with statisticians, health economists, and those with experience of running and reporting trials to Good Clinical Practice standards in collaboration with the Nottingham Clinical Trials Unit.[12] It also has an educational role by running meetings such as the evidence-based update meeting on a different theme each year.

Much of the philosophy and processes for setting up and running an organization like the UKDCTN could be replicated in India, and in time, there will opportunities for such similar organizations to take part in collaborative studies that require very large numbers of patients from all over the world.

**Table 1:** The five national clinical trials currently being run at the centre of evidence-based dermatology

Study acronym	Condition	Interventions	Funding body	Start date
**SINS**	Low risk nodular and superficial basal cell carcinoma	Excisional surgery versus topical imiquimod cream	Cancer Research UK	2004
**PATCH I**	Recurrent cellulitis of the leg	Prophylactic penicillin versus placebo	Action Medical Research	2007
**SWET**	Childhood eczema	Ion exchange water softeners versus standard care	NIHR Health Technology Assessment Programme	2008
**BLISTER**	Bullous pemphigoid	Doxycycline versus oral prednisolone	NIHR Health Technology Assessment Programme	2009
**STOPGAP**	Pyoderma gangrenosum	Oral prednisolone versus oral ciclosporin	NOHR Applied Research Programme Grant	2009

## Step 3: Disseminating Knowledge to a Community of Clinical Users

### Knowledge is worth little unless it is shared and put to good use

Producing lots of information is of little use unless it is filtered, organised, and made available for those working in clinical practice. Such is the purpose of the National Library of Health Skin Disorders Specialist Library – a one stop shop of quality information resources all in one place and kept up-to-date on a daily basis by a full-time information scientist based at our Centre of Evidence-Based Dermatology.[13] At this stage, you may be thinking to yourself, “It is all right for them, they have a Government funded information specialist.” But again, like the Cochrane Library, our resource is completely free for anyone living in India with access to the Internet. The information resources included in the Skin Disorders Library are quality appraised to avoid biased information. The resources are organised into guidance and pathways, evidence sources, reference articles, education and continual professional development, and patient information resources. Special features include guest editorials, an opportunity to submit uncertainties that require research to the Database of Uncertainties of the Effects of Treatments (DUETs),[14] and the annual evidence updates.

### The annual evidence-based updates

The annual evidence updates deserve a special mention since they go beyond just organisation of information. Each year, the four topics of acne, skin cancer, eczema, and psoriasis are searched in detail by our information specialist for newly published systematic reviews or trials. The new resources are then listed according to whether they deal with causes, treatment type, or effects on quality of life. For each update, experienced clinicians then provide a commentary on what the evidence might mean for clinical practice as well as drawing attention to some of the methodological limitations and misleading aspects of the evidence found.[15]

Although the Skin Disorders Specialist Library is produced primarily with UK health care practitioners in mind, it is likely that most of the information will be useful for dermatologists in India. Comprehensive information is also available in the Infectious Diseases Specialist Library for those interested in sexually transmitted diseases.

## How It All Fits Together

**Figure 5:**
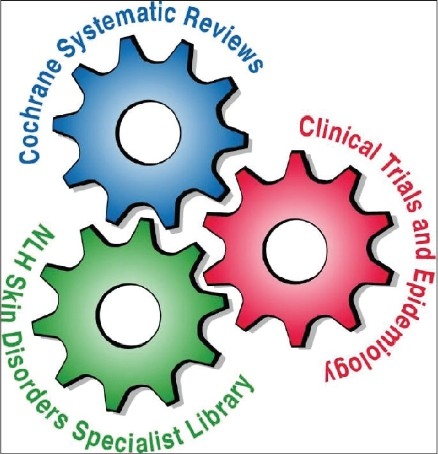
The three cogs of the centre of evidence-based dermatology composed of the Cochrane Skin Group that identifies and prioritises uncertainties, the UK dermatology clinical trials network that then addresses those uncertainties through randomised controlled trials and the skin disorders specialist library which then disseminates the research findings to a community of clinical users

At first sight, the three main activities of the Centre of Evidence-Based Dermatology i.e., the Cochrane Skin Group, the UK Dermatology Clinical Trials Network, and the Skin Disorders Specialist Library may sound like three separate and independent activities. But nothing could be further from the truth because each work aspect drives the other like cogs working together in a machine [Figure 5]. The systematic review cog summarises what is known about current dermatology treatments and highlights research gaps that are then picked up by trials conducted by the UKDCTN cog. Once those trial results are known, they are disseminated to a community of users by the Skin Disorders Library cog, thereby closing the loop of knowledge generation and utilisation. So the work at the Centre all fits beautifully together and is also informed by other projects at the Centre such as epidemiology, development of outcome measures, or the production of decision aids to help doctors and patients choose between difficult treatment options – work that will emerge as part of our recently awarded program grant.

## How You Can Contribute to Promoting Evidence-Based Dermatology

### Systematic reviews

Perhaps the best way that any colleague in India can contribute to evidence-based dermatology is to simply look at and hopefully use the information that our Centre helps to produce. The value of such knowledge is nil unless it used in the clinic. I hope that at least some of our output can be put to good use in clinical practice in India. Even though some of the material is less applicable to skin problems in India because of some differences in the disease spectrum, resources and culture, much of it will be relevant because there are more similarities between people around the world than there are differences. There are some good online resources for learning about evidence-based medicine that are listed on our homepage including dermatology's own www.ebderm.org, which our Centre helps to support. Those who want to do more, please consider joining the Cochrane Skin Group. The Cochrane Skin Group is a truly international group that holds annual meetings and training events.[16] Some will want to join a review team, some might want to help us by refereeing protocols and reviews, and some will end up leading a Cochrane review. True, it is very hard work, as it takes around 2 to 3 years to complete a systematic review, but it is worthwhile because it provides the person with a wonderful grounding in critical appraisal skills.

### Clinical trials

Then there are clinical trials. At the moment, the UK Clinical Trials Network is focused on delivering independent trials in the UK, but at some stage in the future we would like to extend collaboration to colleagues across the world if we can find suitable enthusiastic investigators and funding that will make such international studies possible. Perhaps in time, you will develop your own Centre for Evidence-Based Dermatology with a portfolio of independently produced resources that are chosen by you to be relevant to your needs. Good progress in this regard has already been made by Dr. S.R. Narahari and colleagues at the Institute of Applied Dermatology by their approach to researching Ayuervedic medicines in an integrated way.[17] It is my vision that in time, lots of other similar initiatives for developing evidence-based dermatology and conducting high quality trials will spring up all over the world, which will then join together so that collective intelligence is multiplied and shared for the betterment of mankind. One day, I hope to see a lot more cogs all working together in one global machine producing high quality and clinically relevant research information for people with skin disease. May that day come soon.
